# Emerging Use of Social Media in Clinical Urology Practice in the 21st Century: Survey Study

**DOI:** 10.2196/58510

**Published:** 2024-12-16

**Authors:** Mohammed Alfozan, Saad Alshahrani, Raed Alasmi

**Affiliations:** 1 Department of Surgery, College of Medicine, Prince Sattam bin Abdulaziz University, Al-Kharj, 16278, Saudi Arabia, 966 11 588 8888

**Keywords:** delivery of health care, social media, urologists, urology, Saudi Arabia, professional communication, physician behavior

## Abstract

**Background:**

Social media (So-Me) platforms are valuable resources for health care professionals and academics to discover, discuss, and distribute current advances in research and clinical practices, including technology trends.

**Objective:**

This study aims to assess the role of So-Me in urological practice in Saudi Arabia. It explores the influence of digital platforms on patient interaction, professional communication, decision-making, and education.

**Methods:**

The survey was conducted among 145 urologists from July 2021 to July 2022 following institutional review board approval. A questionnaire designed using the SurveyMonkey platform examined urologists’ knowledge of So-Me. The survey was conducted using the CHERRIES (Checklist for Reporting Results of Internet E-Surveys) guidelines and was open for 17 weeks. Data analysis was performed using SPSS 21.0.

**Results:**

Of the 145 participants, 70% (n=102) were Saudi Arabians. The most common age groups were 30‐40 (n=68, 46.8%) and 41‐50 (n=61, 42.2%) years, with a gender distribution of 44.8% (n=65) women and 55.2% (n=80) men. A total of 61.5% (n=89) of urologists reported using So-Me accounts for professional purposes, with 54.9% (n=80) sharing health-related information. Social media enhanced patient connections beyond clinic visits for 55.8% (n=81) of respondents, while 57.2% (n=83) used it to provide educational resources. Additionally, 56.5% (n=82) believed So-Me facilitated patient feedback and improved their practice. In terms of professional communication, 60.6% (n=88) of urologists agreed that So-Me facilitated collaboration with colleagues, while 63.3% (n=92) used it to stay updated on the latest advances in urology. Furthermore, 62% (n=90) followed professional societies or journals on So-Me, and 63.3% (n=92) used it for continuing medical education. A majority (n=94, 64.7%) reported that So-Me influenced treatment decisions based on new research findings, and 85.3% (n=124) learned about novel technologies and treatment options through these platforms. Regression analysis showed a significant positive correlation between gender and social media usage patterns (*R*=0.653, *R*^2^=0.426), indicating that approximately 42.6% of the differences in usage patterns can be attributed to gender. However, the Pearson *χ*^2^ analysis showed that gender did not significantly affect most aspects of social media use, except information sharing and participating in online discussions (both *P*<.05).

**Conclusions:**

This study highlights the widespread use of So-Me among urologists in Saudi Arabia, underscoring its role in enhancing patient interaction, professional development, and clinical decision-making. Strategically designed health care programs using social media could improve and modernize professional and patient-centered care in Saudi Arabia through legislative assistance and guidelines.

## Introduction

Social media (So-Me) platforms are valuable resources for health care professionals and academics, providing opportunities to discover, discuss, and share advances in research, clinical practices, and emerging technology trends. Many potential applications of So-Me have been developed, and journals and researchers are increasingly using it to connect with their end consumers for education and knowledge translation.

Traditionally, urologists have incorporated innovative technology trends such as robotic surgery and laser techniques into their clinical practice and have participated in real-time global interactions through So-Me. These platforms aid in exploring opportunities that facilitate participation in e-conferences, discussions on recent guidelines and scientific publications, and interactions with both fellow health care professionals and patients [1,2]. Social media encompasses various web-based apps featuring user-generated content including microblogging (Twitter), social networking (Facebook and LinkedIn), and photo-video sharing (Instagram, YouTube, Vine) [3].

In a survey on So-Me use in urology, the American Urological Association found a significant rise in So-Me use by urologists, with 74% agreeing that it had an important influence on their practice [4]. LinkedIn (46%), Facebook (93%), Twitter (36%), and Google+ (26%) are the most popular social media sites among urologists [5]. Other So-Me platforms in native languages are being used by urologists from other nations; QQ, We Chat, and RenRen are the most popular So-Me platforms among Chinese urologists [6].

In comparison to consultant urologists in high-income countries, those in low-income countries have reported low engagement and perceived professional use of So-Me. Health care professionals in Saudi Arabia use So-Me for networking (27%), professional education (24%), and health promotion (13%) [7].

Social media use is increasing both among health care professionals and the general public. Globally, the total number of So-Me users on the platforms Twitter, LinkedIn, Instagram, WhatsApp, YouTube, and Facebook are nearly 436 million, 610 million, 1.478 billion, 2 billion, 2.562 billion, and 2.91 billion, respectively, with numbers increasing continuously every year [8]. A survey-based study examining So-Me use among health care professionals found that 42% of the participants identified as passive So-Me users, recognizing advantages such as networking (49%) and staying updated on recent research publications (66%) [9]. In 2022, Turner et al [10] reported that approximately 60% of 115 participants used So-Me for continuous professional development on platforms like Facebook owing to the ease of navigation and accessibility.

Despite the massive penetration of social media platforms, with WhatsApp (73%) being the most popular one [11], there have been few studies on the influence and use of social media by health care professionals in Saudi Arabia. To our knowledge, this is the first study to investigate the role and impact of social media on clinical practices among urologists in Saudi Arabia.

The shift from print to online media has changed how the medical community assesses the influence of scientific literature. Instead of relying solely on the number of citations, newer metrics such as the number of tweets, blog posts, article page views, and downloads now gauge the popularity and impact of a specific publication. This study aims to assess the role of So-Me on urology practices in Saudi Arabia, examining the influence of digital platforms on patient interaction, professional communication, decision-making, and education.

## Methods

### Study Design

This survey study was conducted to identify the correlation between So-Me use, awareness, and attitudes of urologists in Saudi Arabia toward So-Me. The study was conducted from July 2022 to July 2023 with the survey remaining open for 17 weeks and was later closed for participants for data analysis.

### Study Population and Sample

The study included urologists practicing in Saudi Arabia selecting participants from various regions. Participants were residents, associate consultants, and consultant urologists who provided informed consent and filled out the survey form. A total of 200 urologists were recruited for the study using a convenience sampling technique from 220 hospitals across Saudi Arabia.

### Ethical Considerations

This study was approved by the Deanship of Scientific Research—Research Ethics Committee in Health and Science Disciplines at Prince Sattam bin Abdulaziz University (REC-HSD-129-2022), ensuring adherence to ethical standards for human subject research. Informed consent was obtained from all participants, and the original informed consent allowed for secondary analyses without requiring additional consent. Privacy and confidentiality were maintained by anonymizing or deidentifying all data before analysis, with no personally identifiable information included. This study did not include any images that could identify individual participants. Moreover, this study did not involve the collection of data on gender, gender identity, or sex assigned at birth, as these variables were deemed irrelevant to the research objectives. The participants were not compensated.

### Study Tool

After obtaining consent from 170 urologists, a close-ended, self-developed questionnaire comprising personal information, use, awareness, and attitude of urologists toward social media was emailed to the official emails of the willing participants (closed survey). The questionnaire was designed using the SurveyMonkey platform to examine urologist knowledge related to social media use. The survey adhered to the CHERRIES (Checklist for Reporting Results of Internet E-Surveys) checklist.

The CHERRIES checklist includes a questionnaire design that includes all patterns of questions required for the study and consists of multiple methods of providing numeric responses. Our questionnaire was easy to send to the participants through the internet for collecting data, and the participant could fill out a single questionnaire many times. Since the study is based on a closed survey, it is supported by the CHERRIES checklist strategy, which has a specific question-and-answer policy [12].

The survey questionnaire consisted of three sections: (1) respondent demographics, (2) questions related to the use of social media using a 5-point Likert scale (5, strongly agree, to 1, strongly disagree), and (3) questions pertaining to professional communication. A total of 145 urologists filled out the questionnaire and were included in the study. Participants with incomplete responses were excluded, resulting in a response rate of 75.5% in this study.

The value of Cronbach α 0.754 for the reliability of the questionnaire items (n=22) revealed that the questionnaire has internal consistency among all questions.

### Data Analysis

Descriptive statistics, reliability tests, cross-tabulation, *χ*^2^, and regression analyses were conducted using SPSS (version 21.0; IBM Corp).

## Results

The study recruited 145 urologists in Saudi Arabia to identify the emerging use and perception of So-Me. Table 1 displays the baseline characteristics of the participants. The majority of participants were Saudi Arabian nationals (n=102, 70%) and 30% (n=43) were not Saudi Arabian. The majority of participants (n=68, 46.8%) were between the ages of 30-40 years, followed by 42.2% (n=61) who were between the ages of 41-50 years, and 11% (n=16) who were older than 50 years. In terms of gender, 55.1% (n=80) were men, and 44.8% (n=65) were women. Most of the participants (n=59, 40.6%) were residents, while 32.4% (n=47) were registrars/associate consultants and 29.5% (n=39) were consultants. In terms of current practice, the majority of participants (n=87, 60%) were from government hospitals, followed by 26.8% (n=35) from academia and 15.1% (n=23) from private practice.

**Table 1.** Demographic characteristics of urologists.

Variables	Participants (N=145), n (%)
Gender	
Men	80 (55.1)
Women	65 (44.8)
Age (years)	
30-40	68 (46.8)
41-50	61 (42.2)
>50	16 (11.0)
Current job	
Resident	59 (40.6)
Registrar/associate consultant	47 (32.4)
Consultant	39 (26.8)
Current practice	
Academic institutions	35 (24.1)
Government hospital	87 (60.0)
Private clinics	23 (15.8)
Nationality	
Saudi Arabian	102 (70.3)
Non–Saudi Arabian	43 (29.7)

The majority of the participants in this study were urology residents, accounting for 40.6% (n=59) individuals. Additionally, the majority of participants (n=87; 60%) were affiliated with government hospitals, while 35 (26.8%) were from academic institutions and 23 (15.8%) were from private practices.

Table 2 depicts the responses of participants to the questionnaire. Section one contains questions about social media use, and a majority of the participants indicated that they use So-Me for professional purposes (n=79, 54.5%), with 52.3% (n=76) reporting that they share health-related information on their So-Me accounts. Of the 145 participants, 52 stated they engage with patients and colleagues on So-Me, while 61 reported active participation in online discussions related to urology.

**Table 2.** Responses of participants (N=145) to the questionnaire

Questions	Strongly disagree, n (%)	Disagree, n (%)	Neutral, n (%)	Agree, n (%)	Strongly agree, n (%)
Social media usage					
I utilize social media platforms for professional purposes.	16 (11.0)	18 (12.4)	32 (22.1)	66 (45.5)	13 (9.0)
I have separate personal and professional social media accounts.	18 (12.4)	19 (13.1)	32 (22.1)	62 (42.7)	14 (9.6)
I share health care information on social media.	9 (6.2)	16 (11.0)	44 (30.3)	51 (35.1)	25 (17.2)
I engage with patients and colleagues on social media platforms.	16 (11.0)	23 (15.8)	42 (28.9)	52 (35.8)	12 (8.2)
I actively participate in online urology communication or forums.	13 (8.96)	21 (14.8)	30 (20.6)	58 (40.0)	20 (13.7)
Patient interaction					
Social media helps me connect with patients beyond a clinic visit.	12 (8.27)	18 (12.4)	34 (23.4)	58 (40.0)	23 (15.8)
Patients use social media to ask me questions or seek appointment information.	13 (8.9)	17 (11.7)	44 (30.3)	50 (34.4)	21 (14.4)
Social media helps me build trust and close relationships with patients.	12 (8.2)	20 (13.7)	32 (22.1)	60 (41.3)	21 (14.4)
I use social media to provide educational resources to patients.	6 (4.1)	14 (9.6)	42 (28.9)	56 (38.6)	27 (18.6)
Social media facilitates patient feedback and improves my practice.	10 (6.8)	10 (6.8)	43 (29.6)	48 (33.1)	34 (23.4)
Professional communication					
Social media helps me connect and collaborate with other urologists.	8 (5.5)	9 (6.2)	40 (27.5)	56 (38.6)	32 (22.1)
I follow professional societies and journal social media platforms.	8 (5.5)	9 (6.2)	38 (26.2)	52 (35.8)	38 (26.2)
Social media enables me to stay updated on the latest advances in urology.	8 (5.5)	9 (6.2)	36 (24.8)	60 (41.3)	32 (22.1)
I engage in online discussions and share knowledge with other urologists.	8 (5.5)	7 (4.8)	44 (30.3)	56 (38.6)	30 (20.6)
Social media helps me participate in continuing medical educational activities.	6 (4.1)	13 (8.9)	34 (23.4)	61 (42.1)	31 (21.3)
Decision-making and education					
Social media influences my treatment decisions based on new research findings.	4 (2.7)	11 (7.5)	36 (24.8)	51 (35.1)	43 (29.6)
I use social media to learn about new technology and treatment options.	5 (3.4)	8 (5.5)	48 (33.1)	80 (55.2)	44 (30.3)
Social media platforms are reliable sources of educational content for urologists.	7 (4.8)	6 (4.1)	43 (29.6)	56 (38.6)	32 (22.1)
I am concerned about the accuracy and misinformation on social media regarding urology.	5 (3.4)	6 (4.1)	35 (24.1)	64 (44.1)	35 (24.1)
I would recommend using social media as a professional developmental tool for another urologist.	6 (4.1)	5 (3.4)	43 (29.6)	62 (42.7)	29 (20.0)

The second section of the questionnaire focused on patient interaction. Most participants agreed that social media helps them connect with patients beyond clinic visits (n=81, 55.8%). Additionally, 48.8% (n=71) stated that patients use social media to ask questions or seek appointment-related information. A total of 55.7% (n=81) agreed that social media helps them build trust and strong relationships with patients, while 57.2% (n=83) agreed that they use social media to provide educational resources. Furthermore, 56.5% (n=82) noted that social media facilitates patient feedback and improves their practices.

The third section focused on professional communication. A total of 60.6% (n=88) agreed that social media helps them connect and collaborate with other urologists. Additionally, 62% (n=90) stated that they follow professional societies and journal accounts, while 63.3% (n=92) agreed that social media enables them to stay updated on the latest advances in urology. A total of 59.2% (n=86) agreed that they engage in online discussions to share knowledge with other urologists, and 63.3% (n=92) stated that social media helps them participate in continuing medical education.

The last section contained questions related to their education and decision-making. Out of 145, 64.7% (n=94) agreed that social media influences their treatment decisions based on novel research findings. A significant 85.3% (n=124) agreed that social media helps them learn about new technologies and treatment options, and 60.6% (n=88) stated that the content on social media is reliable. Additionally, 62.7% (n=91) agreed they would recommend using social media as a developmental tool for other urologists.

A Pearson *χ*^2^ test was performed to examine gender-based differences (Table 3). This table provides insights into the perceptions and behaviors of urologists regarding So-Me use, patient interaction, professional communication, decision-making, and education, based on the statistical analysis of the survey data. Overall, findings show that gender did not play a significant role in most major aspects of So-Me use. However, sharing information on social media, actively participating in discussions and forums related to urology, and engaging in continuous medical education activities were positively associated with gender (all *P*<.05).

**Table 3.** The outcome of the Pearson *χ*^2^ analysis.

Questions	Asymptotic significance (2-sided) *P* value
Social media usage	
I utilize social media platforms for professional purposes.	.83
I have separate personal and professional social media accounts.	.01
I share healthcare information on social media.	.01
I engage with patients and colleagues on social media platforms.	.08
I actively participate in online urology communication or forums.	.03
Patient interaction	
Social media helps me connect with patients beyond a clinic visit.	.13
Patients use social media to ask me questions or seek appointment information.	.15
Social media helps me build trust and close relationships with patients.	.80
I use social media to provide educational resources to patients.	.77
Social media facilitates patient feedback and improves my practice.	.59
Professional communication	
Social media helps me connect and collaborate with other urologists.	.70
I follow professional societies and journal social media platforms.	.70
Social media enables me to stay updated on the latest advances in urology.	.95
I engage in online discussions and share knowledge with other urologists.	.87
Social media helps me participate in continuing medical educational activities.	.007
Decision-making and education	
Social media influences my treatment decisions based on new research findings.	.18
I use social media to learn about new technology and treatment options.	.15
Social media platforms are reliable sources of educational content for urologists.	.99
I am concerned about the accuracy and misinformation and social media regarding urology.	.32
I would recommend using social media as a professional developmental tool for another urologist.	.65

A regression analysis was performed to examine the relationship between gender (independent variable) and various questionnaire items related to social media usage, patient interaction, professional communication, decision-making, and education (dependent variables) among urologists. The correlation coefficient (*R*) of 0.653 suggests a moderately strong positive linear relationship between gender and the combined set of questionnaire items. The coefficient of determination (*R*^2^) is 0.426, suggesting that approximately 42.6% of the variability in questionnaire responses is explained by gender. After adjusting for the complexity of the model, the adjusted *R*^2^ value decreases slightly to 0.333. This adjustment accounts for the number of questionnaire items included in the analysis and provides a more conservative estimate of the proportion of variance explained. The SE of the estimate (0.408) reflects the mean deviation of observed responses and the regression line, indicating the accuracy of predictions made by the model. These findings suggest that gender may significantly influence the attitudes and behaviors of urologists related to So-Me use, patient interaction, professional communication, decision-making, and education within the field.

Table 4 presents the results of the model significance test, which assesses the overall significance of the regression model. The regression model is statistically significant as indicated by *P*<.001, suggesting that the relationship between the independent and dependent variables is not due to random chance. Therefore, the model effectively explains a significant portion of the variability in the dependent variable.

**Table 4.** Result of model significance test.

		Sum of squares	*df*	Mean square	*F* test (*df*)	*P* value
Model 1					4.569	.001
Regression		15.200	20	0.760		
Residual		20.460	123	0.166		
Total		35.660	143	—^a^		

a Not applicable.

## Discussion

This study identified how digital platforms influence patient interaction, professional communication, decision-making, and education and urologist practices in Saudi Arabia. More than 50% of urologists use So-Me professionally to study procedures, share information, communicate with other urologists and patients, and engage in online discussions about medical education. So-Me platforms have facilitated a strong connection among health care providers.

The easy availability and accessibility of these platforms can solve several critical issues in urology care, with facilities enhancing over time. According to the participating urologists, So-Me use is associated with improved patient care and training quality, participation in social events, frequent use in daily professional practice, and increased public awareness.

Bibault et al [13] emphasized that So-Me offers a valuable opportunity to learn about patients’ experiences toward the treatment, enabling health care providers to make improvements accordingly. From the patient’s perspective, interaction with health care providers on So-Me positively impacts the patient-provider relationship, offering convenient access to health care providers and consultations, and information related to their health issues. Patients often self-educate by following their health care providers on So-Me, which strengthens the patient-consultant relationship [14].

An integrated review highlighted the role of So-Me in the development of virtual communities to facilitate knowledge sharing, evidence-informed practice, and professional networking among health care providers. The findings suggested emerging evidence for the development of virtual communities among health care professionals that use So-Me for knowledge sharing related to their domains [15]. However, while interactions on So-Me are perceived to enhance care and training quality, integrating this shared information into clinical practice remains a concern. The rapid sharing of findings could lead to the adoption of practices that could cause harm if the evidence later proves to be incorrect, warranting caution in interpreting the new data shared on So-Me and its clinical application [16].

A similar study examined the professional use of So-Me platforms among radiographers. On average, participants spend approximately 2 hours and 23 minutes on So-Me platforms daily, with about 27 minutes dedicated to content relevant to radiographers. Facebook was the most preferred So-Me platform (93%); a majority of radiologists use these platforms for online meetings, conferences, and teaching. This underscores the potential of So-Me in supporting health care professionals [17]. The engagement of urologists through So-Me has provided them with a useful platform for exchanging urology practice-related information. Another study reports that majority of the urologists (81%) primarily used So-Me to watch procedures and lectures, while 65% participated in medical case discussions. In other medical fields, such as cardiovascular practice [18], So-Me has a substantial impact on the cardiology community. However, findings suggest that the professionals should guide this online community rather than be influenced by it, advocating for responsible and active communication on social media. So-Me platform popularity varies globally; for instance, WhatsApp is widely used by health care professionals in Saudi Arabia, while Twitter or X is popular among radiographers in the United Kingdom [19]. Previous studies in the United States found that a person spends an average of about 2 hours per day on So-Me [20], while another study reported 5 hours per week, with 49.5% of the participants using Facebook [9]. An international survey showed that 79% of participants spend at least 1 hour daily on So-Me platforms for professional purposes [21]. So-Me gives a platform for patients and urologists to actively engage in discussions on health care, with So-Me platforms such as Twitter or X being popularly used for medical purposes worldwide [22].

For evidence-based practice, health care professionals require internet and social media access for extracting and sharing information. However, an empirical study found that excessive So-Me use for entertainment and personal networking can disrupt work-life balance and performance due to conflicts between work demands and technology use [18]. In contrast, using So-Me for professional information-sharing decreases psychological strain, highlighting the need to understand the purpose behind So-Me use due to its varying impacts.

This study has limitations, including a limited sample size, which may not represent urologists across Saudi Arabia. Additionally, the responses were subjective given the survey-based nature of data collection. Therefore, future studies with larger sample sizes and more comprehensive questionnaires are needed to capture further details and broader characteristics of So-Me use among urologists.

In conclusion, this study assesses the role of So-Me on urologist practice in Saudi Arabia, specifically examining how digital platforms influence patient interaction, professional communication, decision-making, and education. The findings reveal that a majority of urologists use So-Me professionally for studying procedures, interacting with patients, and engaging in discussions with colleagues. Social media has facilitated greater interactions among health care providers, enabling them to connect with peers and consultants globally. Its easy accessibility and availability have proven beneficial in resolving critical urology-related challenges and improving health care facilities. Additionally, the study underscores the positive impact of So-Me on enhancing patient care, professional engagement, and daily practice. Several urologists in this study recommend So-Me as a valuable tool for professional development and knowledge exchange among colleagues.

## Abbreviations

CHERRIES: Checklist for Reporting Results of Internet E-Surveys
So-Me: social media
